# Nanomaterials and Nanostructures Hand-In-Hand with Biology

**DOI:** 10.3390/nano12142317

**Published:** 2022-07-06

**Authors:** Gonzalo Villaverde, Alejandro Baeza, Sergio Gómez-Graña

**Affiliations:** 1 Departamento de Química en Ciencias Farmaceuticas, Universidad Complutense de Madrid, 28040 Madrid, Spain; gonvilla@ucm.es; 2 Departamento. Materiales y Producción Aeroespacial, ETSI Aeronáutica y del Espacio, Universidad Politécnica de Madrid, 28040 Madrid, Spain; alejandro.baeza@upm.es; 3 CINBIO, Departamento de Química Física, Universidade de Vigo, 36310 Vigo, Spain; 4 Instituto de Investigación Sanitaria Galcia Sur, Hospital Álvaro Cunqueiro, 36213 Vigo, Spain

The nanoparticle’s synthesis had its tipping point at the beginning of the 21st century, opening up the possibility of manufacturing nanoparticles with almost every imaginable shape and size [1]. Thanks to basic research in the synthesis of nanomaterials, they can be applied in many fields, for instance, art repair [2], electronic materials [3], medicine [4,5], or biology [6]. One of the most attractive challenges for researchers, due to its complexity, was the implementation of nanomaterials in biology research.

This Special Issue is focused on “Nanomaterials and nanostructures for Biology” with the aim of covering the state-of-the-art and most recent advances, as well as the new trends towards the application of nanoparticles in biology research. The manuscripts included in this Special Issue are very wide-ranging, being from the synthesis of nanomaterials and nanostructures to be used as Surface Enhancement Raman Scattering (SERS) substrates, to the synthesis of nanoparticles using natural extracts for the treatment of diabetes, or the synthesis of nanoparticles to catalyze reactions of interest.

SERS is a very powerful technique that allows us to quantify and discern between molecules because each molecule has a fingerprint in Raman [7]. The great advantages of SERS mean that thousands of articles are published every year using this technique for different purposes. Focusing on this Special Issue, the authors used silicon nanopillars coated with molybdenum disulfide (MoS_2_) as SERS substrates [8]. The preparation of these substrates is done by thermal growth using a mask, allowing Si cylinders to grow perpendicular to the surface. They are subsequently treated with a molybdenum oxide and sulfurized, obtaining the desired nanostructures coated with a MoS_2_ layer, which enhances the Raman signal of the analytes. These nanostructures were used to detect contaminants, such as rhodamine 6G (R6G). This paper indicates that the Si/MoS_2_ core–shell nanoparticles arrays are excellent SERS substrates with great potential, remaining possible to expand the application of these 2D material core–shell nanostructure to biomolecules detection.

Similarly, another manuscript submitted to this SI, studied the fabrication of membranes using silver nanowires to employed as SERS substrates too [9]. These nanowires were synthesized by electrochemical cycling voltammetry on a cheap and flexible substrate, such as filter paper. In the article, these substrates were used for the detection by SERS spectroscopy of contaminating molecules, such as crystal violet, thiram, sodium perchlorate, or fluoranthene, among others. Here, the authors have reported an easy, cheap, and highly efficient SERS substrate for the detection of chemicals.

Using natural products for the biogenetic synthesis of nanomaterials nowadays is a trend since it has great advantages over the use of toxic molecules [10]. In this sense, the authors have synthesized silver nanoparticles using Phagnalon niveum [11]. After the synthesis and characterization, they have used the nanoparticles for “in vivo” studies with Alloxan-Induced Diabetic Wistar rats to study the antidiabetic effect of Phagnalon niveum silver nanoparticles observing a significant improvement from the beginning of the treatment (1 day) until 3 weeks later, lowering the blood glucose levels. Furthermore, the treatment with silver nanoparticles synthesized with Phagnalon niveum decrease the cholesterol levels (HDL-c, LDL-c, and TGs). This kind of biogenetic synthesis opens a door to target type-2 diabetes.

One of the most extensive uses of metallic nanoparticles is their use in catalysis [12]. Therefore, the use of easily synthesizable, recoverable catalysts with a high recyclability capacity is important. A significant topic in catalysis is the transformation of toxic molecules into others that are less harmful to biological media [13]. Thus, the authors have carried out the “in situ” synthesis of palladium and gold nanoparticles on porous polydimethylsiloxane substrates. These substrates were used for the elimination of the p-nitrophenol contaminant, making the reaction in a continuous flow, and could be considered as an approximation to industrial conditions.

As can be comprehended in the literature and part of the manuscripts of this Special Issue, most of the substrates synthesized using plasmonic nanoparticles are used for the detection of biomolecules or contaminants by SERS spectroscopy. Thanks to these very efficient substrates for SERS, it is possible to decrease the detection limits compared to more conventional analytical techniques. Therefore, one of the biggest challenges of this research line is to improve the quality of biological media by detecting contaminants earlier. Likewise, these high-efficiency SERS substrates could be used for the detection of biomolecules in complex biological media, making them perfect for the detection of pathogenic or carcinogenic agents, increasing the early detection, and, therefore, improving the treatments and the people’s quality of life.

As it was presented, the detection of biomolecules is not the only use of nanoparticles in biology, their implementation in disease treatment or prevention, or even for contaminants removal could be very important. To draw this conclusion, we believe that research on nanoparticles and nanostructures applied to biology must go on to be implemented in our daily life.
